# The Australian MotherSafe enhanced service for nausea and vomiting in pregnancy and hyperemesis gravidarum: a mixed methods study

**DOI:** 10.1186/s12884-026-09356-y

**Published:** 2026-05-29

**Authors:** Pippy Walker, Alexis Turner, Sarah Pont, Penelope Fotheringham, Delwyn Cupitt, Andrew Wilson, Debra Kennedy, Amanda Rush

**Affiliations:** 1 Leeder Centre for Health Policy, Economics & Data, Sydney School of Public Health, Faculty of Medicine and Health, The University of Sydney, Level 3, Moore College (CG2), 1 King Street Newtown, Sydney, NSW 2042 Australia; 2 Royal Hospital for Women, South East Sydney Local Health District, Randwick, NSW 2031 Australia; 3 Sydney Medical School Central, Faculty of Medicine and Health, The University of Sydney, Camperdown, NSW 2050 Australia

**Keywords:** Pregnancy, Nausea, Vomiting, Hyperemesis gravidarum, Telephone counselling

## Abstract

**Background:**

Nausea and vomiting in pregnancy (NVP) and hyperemesis gravidarum (HG) can have significant physical, psychological and economic impacts on affected women and their families. With the onset of severe symptoms usually occurring in the first trimester and women in Australia typically not engaging in regular antenatal care until beyond this period, there is a gap in access to suitable models of care. In 2023, the MotherSafe teratogen information service introduced an enhanced NVG/HG service that provides medication advice for both over the counter and prescribed medications and psychological support in line with NVP/HG clinical guidelines introduced at a similar time. The aim of this study is to describe service use, patient symptoms and acceptability of the MotherSafe enhanced service for women with NVP and HG in New South Wales (NSW) Australia.

**Methods:**

This convergent parallel mixed methods study involved women and family members who contacted the MotherSafe enhanced NVP/HG telephone counselling service from inception (April 2023) to June 2024. Where available, routinely collected service data was analysed to understand service use and changes in NVP symptoms (PUQE-24 score) between the initial and first follow up MotherSafe counselling call (*n* = 389). To understand acceptability of the service, semi-structured interviews were conducted with women that had recently accessed the service (*n* = 11).

**Results:**

During the first 15 months of the enhanced NVP/HG service operation, 1194 initial and 534 follow up MotherSafe counselling calls provided NVP/HG information and advice. Changes in NVP symptoms measured by PUQE-24 scores between initial and follow up call were favourable for women where this data was available (*n* = 389, mean score reduction 2.26, *p < *0.001), and interview participants reported overall positive experiences that met their needs.

**Conclusions:**

The service has the potential to address a health service gap for women with NVP/HG. Further research to more rigorously evaluate change in NVP symptoms following MotherSafe counselling and advice is needed to confirm the effectiveness of the service. Service call numbers and feedback from women suggest that more widespread promotion of the service would support increased reach.

**Supplementary Information:**

The online version contains supplementary material available at 10.1186/s12884-026-09356-y.

## Background

Nausea and vomiting during pregnancy (NVP) affects most pregnancies [1, 2], with severe NVP or hyperemesis gravidarum (HG) reported to affect between 0.3–3.6% of pregnant women [1, 3, 4]. Dehydration, fatigue, difficulty with daily activities and psychological impacts contribute to poor quality of life of affected pregnant women and their families [2, 5, 6]; and has societal implications resulting from productivity losses [7]. Guidelines recommend treating women in the community with oral antiemetics and other symptom management medications [8–10], with fluid resuscitation and parenteral anti-emetic administration required for more severe symptoms [8, 9].

While treatment guidelines are clear, existing antenatal models of care do not support timely access to care. This is evidenced by the knowledge that the onset of NVP or HG usually occurs between four and ten weeks gestation [11], yet only 60% of pregnant women in Australia report attending antenatal care within the first ten weeks of pregnancy [12]. Care does not typically commence until 10–20 weeks gestation, where the vast majority of women are seen under public hospital maternity care, private obstetrician specialist care and midwifery group practice case load care models. This is resulting in a reliance on emergency departments and general practitioners during the peak time for HG symptoms, with little, if any, involvement of midwives [13].

Misalignment also exists between clinical guidelines for NVP/HG and prescribing practices [2, 14–16]. Lack of awareness, hesitation to prescribe and dispense to pregnant women and a need for more readily available information have been reported as health care professional barriers to appropriate care [14, 17]. From the perspective of affected women, there are reports of low treatment uptake [2], and women being denied medications for severe NVP/HG [14]. Many factors influence decisions to take medications during pregnancy, with importance placed on receiving evidence-based safety information [18]. Reports of inadequate treatment prior to hospital admission [19] and impacts on the health system [16, 20] further emphasise the need for equitable access to reputable information and advice concerning NVP/HG.

Digital health initiatives have the potential to improve access to care for pregnant women, given their ability to reduce health inequities and the capability of women in their reproductive years to use technology [21, 22]. A recent review on digital health in pregnancy has highlighted the need for more evidence on the impact of these innovations on patient outcomes, satisfaction and costs [22]. Reviews on HG models of care have identified a need for more robust research to inform health service design for women with NVP/HG [23, 24].

The MotherSafe telephone counselling service [25] was established in 2000 in New South Wales, Australia to provide evidence-based information to consumers and health care providers regarding exposures during preconception, pregnancy and lactation. Information is provided by medical or pharmacy trained health professionals via telephone, with in person or telehealth medical appointments also available for women unable to access a local health care provider for prescriptions. In 2023, MotherSafe received funding to enhance support for NVP/HG. This allowed extended hours of access (to 9 pm) for callers, education and upskilling for the staff in line with clinical guidelines [26] and creation of a new dedicated database for the service. The aim of this study is to describe service use, patient symptoms and acceptability of the MotherSafe enhanced NVP/HG service (‘MotherSafe’).

## Methods

### MotherSafe enhanced NVP/HG service description

The MotherSafe enhanced NVP/HG service provides medication advice for both over the counter and prescribed medications and psychological support in line with new NVP/HG clinical guidelines [26]. All women were offered routine follow up calls at 48–72 h after the initial call (with consent), which were scheduled for willing callers at a time that aligned with clinical need and caller availability. Women were assessed for their NVP symptoms over the previous 24 h with the Pregnancy-Unique Quantification of Emesis and Nausea (PUQE-24) scale [27], and standardised questions assessing their clinical condition. Callers who reported symptoms warranting acute medical review were referred to either their own primary care provider or to a network of NVP/HG mapped referral pathways across the state, dependent on clinical severity. Women unable to access appropriate management in their location could access a telehealth appointment with an obstetrician or obstetric physician in the NVP/HG service. More details about the service are provided in supplementary material (S1).

### Study design

This convergent parallel mixed methods study involved analysis of routinely collected MotherSafe service data on NVP/HG-related calls to assess service usage and patient symptoms. In parallel, semi-structured interviews were conducted with women who had accessed MotherSafe by independent researchers to evaluate service acceptability. Acceptability interviews were chosen over a survey based on previous researcher experience with low response rates among women with NVP/HG and to allow for deeper exploration of women’s perceptions of integral aspects of the service, which could not have been predefined for standardised measurement. Reporting on this study aligns with the RECORD statement for observational studies [28], and the consolidated criteria for reporting qualitative research (COREQ) [29].

### Recruitment

Routinely collected service data included all women who accessed the MotherSafe service for NVP/HG during the first 15 months of the enhanced service's operation (April 2023 to June 2024), with a convenience subsample of these women invited to participate in an interview over a 7-week recruitment period (April—May 2024). At the conclusion of all counselling telephone calls during this period, MotherSafe counsellors (including DK, PF, DC) provided information about the purpose of the research and invited women who were considered physically and mentally able and potentially willing to participate in an interview. Women who indicated that they would like to receive further information about the study were emailed an invitation, which included a participant information sheet and a link to an online consent form hosted in REDCap [30]. Researchers (AR, PW) arranged interviews directly with consenting women via email, and recruitment continued until theme saturation was achieved with a minimum recruitment sample set at 10.

### Data collection

For each call counsellors (including DC, PF and DK) asked callers to provide basic demographic information (e.g., gestation, whether they were Australian Aboriginal or Torres Strait Islander and/or from a culturally and linguistically diverse background), postcode, reason/s for the call, and self-reported PUQE-24 score. Any information voluntary provided by callers was recorded in the MotherSafe database. Due to a database upgrade after the commencement of the enhanced NVP/HG service, the total number of initial calls and follow up calls could be extracted from the database for the period of interest, however not the total number of calls per caller.

The PUQE-24 is a clinical assessment tool that quantifies the severity of nausea and vomiting in pregnancy [27, 31], where a higher score indicates more severe illness: mild ≤ 6; moderate 7 to 12; severe ≥ 13. The PUQE-24 includes three questions, which address time spent feeling nauseous or sick, and number of episodes of vomiting and retching, respectively, in the past 24 h. PUQE-24 scores recorded at initial (pre) and first follow up (post) MotherSafe call were extracted from the MotherSafe database for analysis for all women with at least one follow-up call during the data collection period (April 2023 – June 2024).

An observed effect size from an unpublished quasi-experimental study evaluating the change in PUQE-24 score among women attending a specialist HG clinic [32] estimated that a sample size of 26 pairs of pre and post PUQE-24 scores would be required to achieve a power of 80% and a two sided significance of 5%, assuming that 94% and 61% of the pairs have a moderate or severe score pre- and post- intervention respectively.

Semi-structured interviews were conducted using an interview guide developed by lead researchers (PW, AR) (S2). The interview guide was informed by the theoretical framework of acceptability [33] and other evaluations of patient experiences with maternity care [34, 35]. Interviews were conducted by two female researchers with experience in conducting mixed methods research (PW, AR) either via Zoom or telephone. All interviews were audio recorded and professionally transcribed. All participants were offered the opportunity to review their transcript for comment or correction; however, no feedback was received.

### Data analysis and reporting

Service data was cleaned and analysed to report descriptive statistics on service reach. The dataset was cleaned to remove duplicate patient visit records (*n* = 6) and patients with a missing baseline PUQE score (*n* = 3). To investigate the change in nausea and vomiting symptoms, available initial and first MotherSafe follow up call PUQE-24 score data were analysed as continuous data using a paired t-test, and as dichotomous data using McNemar’s test for PUQE-24 scores that were (1) moderate or severe or (2) severe at initial and follow up counselling calls.

Interview transcripts were analysed deductively using thematic analysis [36] informed by the framework of acceptability [33]. An initial coding framework was established by three researchers (AT, PW, AR) after each had coded the first transcript. Coding was then completed for all transcripts by one analyst (AT) using NVivo qualitative data analysis software [37], and then validated by two other researchers (PW, AR).

## Results

### Calls to MotherSafe for NVP/HG

**Fig. 1:**
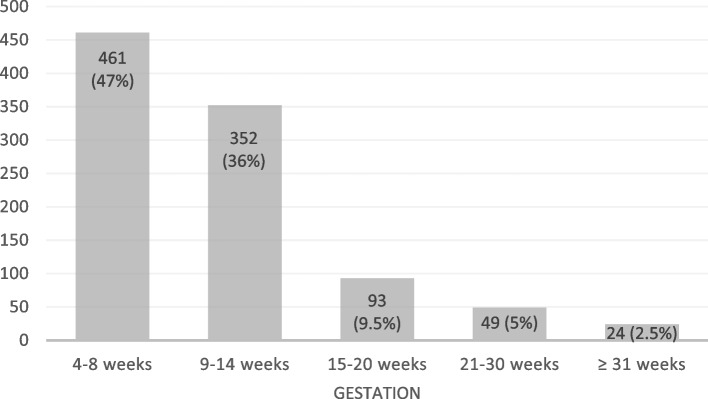
Gestation of women calling MotherSafe for support with NVP/HG (*n* = 979, with gestation not recorded for 24 (2.4%) of initial calls)

From April 2023 to June 2024, MotherSafe counsellors answered a total of 1194 initial calls for advice on NVP/HG and made 534 follow up calls. A description of the 1194 initial callers, of which 1003 (84%) were from pregnant women with NVP/HG is presented in Table 1. Most women were in their first trimester at the time of their first call to MotherSafe, with 47% (*n* = 461) between 4–8 weeks and 36% (*n* = 352) between 9–14 weeks gestation (Fig. 1). The most common reasons for calling MotherSafe reported by women with NVP/HG were to discuss medications that had been prescribed by a health professional and/or to seek further clarifications after obtaining advice from elsewhere (Table 2).

**Table 1:** Description of initial callers for advice on NVP/HG

Characteristic		n (%)
Type of caller (*n* = 1194)	Pregnant women	1003 (84.0%)
	Family members or friends of a women with NVP/HG	85 (7.1%)
	Health care professionals	106 (8.9%)
Caller location [38] of pregnant women and family member/friend callers (*n* = 1056; *n* = 32 (2.9%) data missing)	Major Australian city	911 (86.3%)
	Inner regional area	122 (11.6%)
	Outer regional area	16 (1.5%)
	Remote area	4 (0.4%)
	Very remote area	0
	Overseas	3 (0.3%)
Cultural background of pregnant women callers (*n* = 1003)	Culturally and linguistically diverse background	122 (12.1%)
	Aboriginal or Torres Strait Islander	17 (1.7%)
	Neither culturally and linguistically diverse or Aboriginal or Torres Strait Islander	864 (86.1%)
NVP severity (PUQE-24 Score) of pregnant women callers (*n* = 847; *n* = 156 (15.6%) data missing)	Mild NVP (PUQE-24 score ≤ 6/15)	80 (9.4%)
	Moderate NVP (PUQE-24 score 7–12/15)	576 (68.0%)
	Severe NVP (PUQE-24 score ≥ 13/15)	191 (22.6%)

**Table 2:** Reason for MotherSafe call from women with NVP/HG (*n* = 1003)

Reason for call	n	%
Discuss medication prescribed by health professional or concerned with medication prescribed	208	20.7%
Seeking further advice after getting advice from elsewhere	199	19.8%
Other (e.g., called for another reason/medication and received counselling on NVP/HG)	131	13.1%
Concerned with pregnancy symptoms, not reached out to health professional	117	11.7%
Conflicting advice from others or internet	27	2.7%
Concerned that they have NVP/HG and want to discuss medications available for treatment of NVP/HG	19	1.9%
Experienced refusal to prescribe by doctor or refusal to dispense/supply by pharmacist for NVP/HG medication	12	1.2%
Reason not recorded	290	28.9%

### Changes in NVP symptoms (PUQE-24 scores) between initial and first follow up MotherSafe call

As shown in Table 1, a PUQE-24 score was recorded for 847 (84.4%) of initial calls from pregnant women with NVP/HG. Of the 534 follow up calls that were made, the MotherSafe database recorded a PUQE-24 score at both initial and first follow up telephone call for a total of 389 women (73% of all follow up calls). There was an average of 4 days separating these initial and follow up calls (range 0—26 days), and median gestation at initial call was 8 weeks (*n* = 386; range 4–35 weeks). Upon initial call, 368 (94.6%) women had a moderate or severe PUQE-24 score (score ≥ 7/15), with 111 (28.5%) scoring as having severe NVP symptoms. Upon follow up, 275 (70.7%) of women had a moderate or severe PUQE-24 score, with 42 (10.8%) having severe symptoms. There was a significant reduction in PUQE-24 scores between initial and follow up counselling calls, with a mean score reduction of 2.26 (mean initial call score = 10.44, SD = 2.78; mean follow up call score = 8.19, SD = 2.99; t(388) = 15.09, *p < *0.001 (two-sided)), and medium effect size (d = 0.77). When analysing improvements only among women that had a moderate or severe PUQE-24 at the initial call (score ≥ 7/15) (*n* = 368), the effect size was large (d = 0.82) with a mean score reduction of 2.41 (mean initial call score = 10.72, SD = 2.57; mean follow up call score = 8.31, SD = 3.01; t(367) = 15.75, *p < *0.001 (two-sided)).

The proportion of women who no longer had a moderate or severe PUQE-24 score at follow up (*n* = 98/389, 25%) was significantly greater to the proportion of women who developed moderate or severe NVP symptoms (*n* = 5/389, 1%) (X^2(1)^ = 82.18, *p < *0.001). Similarly, the proportion of women who initially had, but then did not report, severe NVP symptoms at follow up (*n* = 80/389, 21%) was significantly greater to the proportion of women who developed severe symptoms (*n* = 11/389, 3%) (X^2(1)^ = 50.81, *p < *0.001) (Table 3).

**Table 3:** Comparison of proportion of women that had either moderate or severe NVP symptoms, and severe NVP symptoms, at initial and follow up MotherSafe call (*n* = 389)

	**Moderate or Severe NVP symptoms (PUQE-24 Score ≥ 7/15)**		**Severe NVP symptoms** **(PUQE-24 Score ≥ 13/15)**	
	**Follow up call**		**Follow up call**	
Initial Call	No	Yes	No	Yes
No	16	5	267	11
Yes	98	270	80	31
McNemar test asymptotic *p*-value	< 0.001		< 0.001	

### Service acceptability

#### Description of participants and MotherSafe counselling sought and received

Of 75 women who were informed about the opportunity to participate in an interview, 53 agreed to receive further information, 14 signed consent forms, and researchers scheduled an interview with 11 women. The average interview length was 20 min (range 11 to 31 min). One woman opted to participate in the interview alongside her partner, who had been the primary communicator with MotherSafe.

Seven (64%) participants lived in a metropolitan area and 4 (36%) lived in a regional area. All women were pregnant at the time of interview, with six (55%) indicating that they were experiencing their second or later pregnancy. Eight (73%) women were in their first trimester (7–12 weeks gestation), and three (27%) were in their second trimester (15, 18 and 20 weeks) at the time of interview. All women described symptoms of moderate to severe NVP or HG (e.g., hospitalisations, requiring IV fluids, daily and constant nausea and/or vomiting), with four (36%) women indicating that they had experienced similar symptoms in a previous pregnancy. Upon contacting MotherSafe, seven (64%) women indicated that they were already taking medications for NVP, however of these only four had been advised by a health care professional to do so. Others either took over the counter medications or prescriptions from previous pregnancies or family members. Another participant had been prescribed medication but was advised by their GP to check with MotherSafe before commencing, and some participants indicated that they were not taking all medications that they had been prescribed by other practitioners as they were unsure of the safety or correct dosages.

Reasons for women calling MotherSafe were reported, including a management plan due to current or previously poorly managed HG (*n* = 3), to ask about overall safety, interactions, appropriate dosages or duration of use of medications being taken or prescribed for NVP (*n* = 6), or for advice on the safety of other medications unrelated to NVP in pregnancy (*n* = 2). Women reported receiving over and above the information that was initially sought, describing a comprehensive and holistic assessment with personalised advice and support. Advice provided included the addition or removal of medications, tweaking of dosages and timing, liaison with GPs or other health care professionals involved in their care to discuss their care plan, education and reassurance about the safety of medications during pregnancy and ongoing assessment of severity of symptoms including psychosocial impacts of NVP and adjustments to recommendations accordingly. All women indicated that they had been offered a follow up phone call with MotherSafe.

Feedback from women about MotherSafe reflected four key domains: (1) awareness and understanding of the service, (2) access, (3) recipient appraisal, and (4) opportunities for service improvement.

#### Awareness and understanding of the service

Women reported learning about the MotherSafe service through family, friends or their own research (*n* = 3; 27%). Another two (18%) indicated they were already aware of the service through their vocation (nurse and midwife) and six women (55%) had been informed by a medical professional (GP, hospital doctor, obstetrician, midwife). Three participants (27%) indicated that they were not aware of the NVP/HG service until they had called MotherSafe for another reason and heard the telephone menu options for NVP/HG support.

*“We got pregnant on the second IUI and that doctor gave me the MotherSafe number. And she just wanted me to check the medications I was on... And I just was listening to the press 1 for this or whatever, and they said, press 1 for nausea and vomiting. And I thought, oh. I was like, that's what I need.”* – Pt 11

The type of information, support and care that could be provided by the service was reportedly not clear until initial contact with the service. Three women (27%) reported some level of hesitation about making initial contact with MotherSafe, due to previous negative experiences with other health care providers, uncertainty about the type of health care professional that they would be speaking to, and for two women an assumption that this ‘counselling service’ was likely to be focused on providing counselling or support for their mental and emotional health, rather than information and education.

*“I did [have reservations about contacting MotherSafe]. …Because I thought — when I looked at it online that — it was just a counselling service. And I've done counselling in the past and it's been really helpful, but I just wasn't in a place where I could sit on the phone and talk to someone about my feelings. Because I couldn't even sit upright.”* – Pt 10

#### Access

Women spoke favourably about being able to access the service via telephone, particularly if they were still working in their first trimester or if they were not well enough to physically get to their GP.

*“Getting to the GP was awful because I couldn't get in the car; getting in the car was horrible. My husband was having to take mornings off work to get me to the doctor because I couldn't drive. And just being able to have someone ring me on the phone and do this from home when I was so sick, I cannot explain it. It honestly has brought me back to life.”* – Pt 10

Being able to call MotherSafe outside of business hours was also a favourable option, rather than needing to attend an in-person clinic during the day when feeling unwell. Calling after hours also allowed women to have their significant other present for the call, which for some was needed to ensure all the advice could be understood and actioned.

*“I wouldn’t call them if I was working. So having that afterhours is really good. But when [counsellor name] called me for the follow up, she called me at 7.00 pm like we had arranged. … It was great to be able to talk to her at night.”* – Pt 5

Women spoke positively about the MotherSafe service being free of charge and how this increased access to care, particularly for those that reported difficulty with accessing their GP.

*“Well to be honest with you, … we didn’t know if it was going to cost us or not. We just have to do it. …. But I mean the fact that it doesn’t cost us anything and that it’s a free service available to anyone, that’s incredible.” –* Partner of Pt 4

For those who had to wait to speak with a counsellor, there was acknowledgement that the length of the wait time reflected the high quality of the service and the length of their own subsequent telephone call.

*“I was a little bit frustrated. The only downside was the wait time. Like, I was on hold for a while, but then when I actually got through, and recognising how comprehensive and thorough she was with me, I was like, well that makes sense…”* – Pt 7

#### Recipient appraisal

Interviewees indicated that they were satisfied and often thankful for the counselling that they received. Women were unanimous in their reports of the positive impact that the MotherSafe service had on their pregnancy, including their mental and physical wellbeing and overall quality of life. The value of this service was implicit in women’s reflections on what MotherSafe was able to offer them compared to their experience with other health care professionals.

*“They see you afterhours. That’s super-helpful because obviously you can’t call your obstetrician every two minutes. And there’s not really anyone else you can call for this kind of thing, especially those who know intricate detail about HG. Often you just tell someone you’ve got pregnancy sickness. Oh yeah, that’s normal. But not when you’re vomiting five or six times a day and not keeping a single thing down for weeks.” – Participant 4*

*“And it was actually the MotherSafe counsellor that figured out that it was the Maxolon that was making me depressed. She was the first person who mentioned it. And then after she mentioned it I stopped it. But again, the hospital said, I think you should keep taking it. And I was like, but it’s such a simple swap to Stemetil. And instead of being depressed and unable to cope, I was like – do you know what I mean. Yeah, it was just brilliant.” – Participant 3*

Aside from the impact of the advice women received on their health and wellbeing, women also reported several aspects of the service that added to their positive experience. This included feeling heard and having their symptoms validated by counsellors who actively listened to their concerns and demonstrated empathy and understanding of what they were experiencing. One participant also reported that having a service for women run by women made it a more attractive service and comfortable experience.

*“…[the counsellor was] non-judgmental. Like I said, she came from such a place of curiosity around my experiences, which was really nice as well. … it just felt very validated, reassuring, person-centred, and holistic would be how I'd describe it.”* – Pt 7

*“… the other thing that's really attractive about it is it's women... you just feel a bit more understood … just being able to sit in front of a woman on a video call and say, I'm constipated, I’m bloating … made me feel a whole lot more comfortable.”* – Pt 10

Women also commented on the ease with which they could engage with different counsellors for subsequent follow up calls without needing to retell their story, indicating that counsellors referred to notes on what had been discussed previously and that this did not impact the quality of care they received.

*“Yeah, I think I mostly talked to different people. I got the same person a couple of times, but it wasn't an issue. The information was pretty same-same across the whole thing.”* – Pt 11

The offering of follow up calls by counsellors was valued by women, who appreciated MotherSafe taking on the administrative burden to follow up. In some instances, symptoms had worsened and this follow up call provided an opportunity to revise the treatment plan, which resulted in improvements in their health and wellbeing.

*“…the other strength, I think, is that follow-up call. … And I think for a service that's obviously so popular and busy, to go make that conscious effort just to check in and make sure everything was going on track was really valuable.”* – Pt 7

The reputation of the longstanding MotherSafe service for their expertise in exposures during pregnancy was valued by and reported by women as a facilitating factor in their care. There were circumstances reported where women were seeking to confirm the advice provided by their GP upon request of the GP before filling a script, and also circumstances where women could take advice from MotherSafe to their GP or other health care provider who were then willing to prescribe according to the plan advised by MotherSafe.

*“And because I could say it came from MotherSafe, he [obstetrician] just straightaway accepted it. And other doctors as well that I've mentioned, as soon as I said MotherSafe, they were like, oh, okay.”* – Pt 11

Following medication advice provided by the service, several interviewees described the service as life changing.

*“Honestly, I can say that if it wasn’t for MotherSafe I don’t think I would be where I am now. They have been an absolute lifesaver. …She [MotherSafe counsellor] understood and she was willing to help. She didn’t make me feel shut out. She …genuinely cared and I only just spoke to her for the first time and I already felt so reassured just from speaking from the first phone call.”* – Participant 1

#### Opportunities for service improvement

Four women highlighted the need for more widespread promotion of the service, due to their lack of knowledge of the service prior to requiring support for their HG.

*“… maybe if there can be some improvement in the promotion of it [MotherSafe] …. I don't know whether maybe pharmacist is a good avenue because I had gone and seen them and they're like, yeah, there's nothing you can take...”* – Pt 18

Another interviewee indicated that it would be helpful if MotherSafe counsellors could actively refer women directly to hospital-based services to negate the need for additional health provider consultations.

*“… because it's just at the Royal Women's Hospital, it would be great if it was a separate service that could sort of tap into different hospitals…For example, they thought I needed fluids and to go into the hospital, but at the time, it was just too hard. … Because then I have to go get a GP referral and then present myself [to hospital] and then it's a bit more of a hassle”* – Pt 11

## Discussion

This study describes initial service use, patient symptoms and acceptability of the MotherSafe enhanced NVP/HG service. Findings have highlighted that there is potential to help more women to overcome barriers to equitable access to support for NVP/HG and that further research to evaluate the effectiveness of the service is warranted.

Women raised the need for better promotion and communication on service offerings, to minimise any potential user hesitation and promote more widespread access of the service. The potential for broader service use is evidenced by current usage data, where MotherSafe supported approximately 200 women with severe NVP/HG over twelve months, in contrast to an estimated prevalence of HG in NSW of between 271 to 3255 women each year [1, 39]. Furthermore, the sample in this study overrepresents women in major cities (86% compared with 77% of births in NSW), suggesting that underutilisation of the service is primarily in inner regional, outer regional, remote and very remote areas [40].

Baseline PUQE-24 scores in this study are comparable with those reported in literature on another similar NVP/HG support service. The Canadian Motherisk service [41] has previously reported between 90 and 95% of initial callers having a moderate or severe PUQE-24 score respectively, similar to our reported 91% (with between 12% and 17.8% of Motherisk callers and 23% of MotherSafe callers having severe NVP/HG) [42, 43]. Gestation at the time of initial call (8 weeks) was also similar, with the Motherisk service reporting an average gestation of 9 weeks [42].

This study addresses a gap in the literature for digital health interventions that support women with NVP. Other digital intervention studies focused on supporting pregnant women with NVP that included outcomes measures are a pharmacist consultation service [44] and a mobile application [45]. Recognising that both studies recruited a smaller proportion of women with moderate or severe NVP symptoms, neither intervention demonstrated improvements in NVP symptoms or quality of life. Furthermore, the pharmacist consultation was delivered in person for just over one third (35%) of women receiving the intervention, rather than telephone support [44]. These findings justify the need for more robust evaluation of digital health services supporting women with NVP/HG.

With existing literature, this study confirms the need for, and the potential of the MotherSafe service to support pregnant women suffering from NVP. It also supports the rationale for further investment in service promotion. Efforts should focus on women in very early pregnancy or pre-pregnancy, and include community pharmacies since van Vliet et al. [46] has reported a preference for early intervention by women with HG and difficulties with accessing a GP or midwife. Information about the service should also emphasise the free of charge nature of the telephone service, given women without private health insurance have been shown to be less likely to access care for NVP [47].

Interviewing 11 women in this study and in other similar studies was sufficient to evaluate service acceptability [46, 48, 49]. Similar to this study, within this literature the importance of patient-centred care involving both physical and psychological support for women with HG is evident [46, 48–50].

To further support biopsychosocial care, more seamless linkage with multidisciplinary providers is needed. Whilst MotherSafe have established referral pathways across the state, and there are examples of effective care navigation at a local health system level [50], some HG models of care still require GP referral or presentation to the emergency department for initial assessment. Given emergency department presentations are a current health system-level challenge [19, 51], and a woman in this study reported a need for easier access to hospital based care, further health system improvements are needed to support more seamless transitions between services and providers.

This is the first evaluation of an NVP/HG telephone counselling service that reports on patient symptoms and experiences. A few trials evaluated telephone support for women with NVP in developing countries [52–55], however evaluations of existing telephone counselling services for NVP to date [42, 43, 56–60] and other data published on the MotherSafe service [61–66] have focused on service use without including patient symptoms and experiences.

This study presents the largest sample of paired PUQE-24 score data on NVP/HG models of care and services [24]. The sample size (*n* = 389) well exceeds the minimum 26 pairs required to detect a significant difference reported by O’Brien et al. [32], with our findings similar to those used to estimate their sample size (94% reducing to 61%, compared to our finding of 94.6% reducing to 70.7% of women with a moderate or severe PUQE-24 score). Our findings are therefore likely sufficiently powered to detect a statistically significant difference in initial and follow up PUQE-24 scores.

Due to the absence of a control group in this study, we cannot ascertain causality and cannot determine that changes in NVP symptoms were not also influenced by other sources of health advice or a natural resolution of NVP symptoms, which typically resolve by 20 weeks gestation [11]. The short duration between initial and follow up calls (average 4 days) and early gestation of women at their initial call (median 8 weeks) however, limits the likelihood of these effect modifiers respectively and suggests that advice provided by MotherSafe and subsequent action taken by women with NVP (e.g., commencing or adjusting medication) have supported the improvement in symptoms for women where data were available. Whilst the inclusion of a control group was outside the scope of this analysis of routinely collected data, future research should seek to confirm the findings of this study to understand the effectiveness of the service on improving NVP symptoms.

There were incomplete data identified for the following voluntarily provided information: caller demographics, gestation, and reason for calling. We were also unable to determine if there were incomplete data at the follow up PUQE-24 score timepoint, as only data on the total number of follow up calls, not the number of women who received a follow-up call could be extracted from the MotherSafe database. A PUQE-24 score was available for 389/534 (73%) follow up phone calls. Whilst it is likely that the remaining 145 follow up calls were for women with NVP/HG that had already received their first initial follow up call, it is possible that some of these calls were initial follow up calls that did not have a PUQE-24 score recorded. We are also unable to postulate how many and why some women did not receive a follow up call, however note that women with milder symptoms at the initial call may be less likely to require a follow-up call. This is evidenced by the difference in the proportion of women with severe symptoms at initial call for those that received a follow up call (28.5%) compared to all of those with an initial PUQE-24 score recorded (22.6%). As such, the sample may overrepresent women with more severe symptoms, limiting the generalisability of the findings.

For the qualitative study findings, social desirability bias [67] may have influenced recruitment, participation and reported acceptability due to the personal and private nature of pregnancy experiences, the effects of NVP/HG on wellbeing, and the positive impacts on wellbeing associated with effective medication management and psychosocial support [44, 55]. To minimise this bias, interviews were conducted by independent researchers, however women that did not experience an improvement in symptoms may not have been well enough to participate. Reassuringly, data triangulation within our study suggests a low risk of such bias as qualitative findings align with improvements in NVP symptoms. Future evaluations should include the collection of patient service satisfaction scores from all callers to negate any possible recruitment bias on service acceptability.

Whilst the MotherSafe service provides information and support to both consumers and health care professionals, this study did not evaluate acceptability to health care professionals. With access to information reported as a key barrier to providing care [17], and evidence for telephone support for general practitioners reducing specialist and emergency department referrals [68], the impact of this service on addressing needs of health care professionals should be prioritised for future evaluations.

## Conclusions

The Australian MotherSafe enhanced NVP/HG service has the potential to fill a critical health service gap and provide equitable access to care for affected pregnant women. This study provides the first indication of the acceptability of a dedicated telephone counselling service, and justifies the need for further investigation into the impact on NVP symptoms. More widespread promotion to both consumers and health care professionals may enable increased service use.

## Supplementary Information

Supplementary Material 1: The MotherSafe enhanced NVP/HG Service.

Supplementary Material 2: Patient experience and acceptability interview guide.

## Data Availability

Quantitative datasets analysed for this study are available from the corresponding author on reasonable request. Due to ethical concerns, interview transcripts cannot be shared.

## Abbreviations

HG: Hyperemesis gravidarum
NVP: Nausea and vomiting in pregnancy
PUQE-24: Pregnancy-unique quantification of emesis and nausea scale

## References

[1] Einarson TR, Piwko C, Koren G. Quantifying the global rates of nausea and vomiting of pregnancy: a meta analysis. J Popul Ther Clin Pharmacol. 2013;20(2):e171–83. 23863575

[2] Tan A, Lowe S, Henry A. Nausea and vomiting of pregnancy: effects on quality of life and day-to-day function. Aust N Z J Obstet Gynaecol. 2018;58:278–90. 28949009 10.1111/ajo.12714

[3] Nurmi M, et al. Incidence and risk factors of hyperemesis gravidarum: a national register-based study in Finland, 2005–2017. Acta Obstet Gynecol Scand. 2020;99(8):1003–13. 32030718 10.1111/aogs.13820

[4] Pont S, et al. Risk factors and recurrence of hyperemesis gravidarum: a population-based record linkage cohort study. Acta Obstet Gynecol Scand. 2024;103(12):2392–400. 10.1111/aogs.14966. 10.1111/aogs.14966 PMC11610008 39258527

[5] Bai G, et al. Associations between nausea, vomiting, fatigue and health-related quality of life of women in early pregnancy: the Generation R Study. PLoS ONE. 2016;11(11):e0166133. 27814390 10.1371/journal.pone.0166133 PMC5096665

[6] Heitmann K, et al. The burden of nausea and vomiting during pregnancy: severe impacts on quality of life, daily life functioning and willingness to become pregnant again - results from a cross-sectional study. BMC Pregnancy Childbirth. 2017;17(1):75. 28241811 10.1186/s12884-017-1249-0 PMC5329925

[7] Piwko C, et al. The weekly cost of nausea and vomiting of pregnancy for women calling the Toronto Motherisk program. Curr Med Res Opin. 2007;23(4):833–40. 17407640 10.1185/030079907x178739

[8] Nelson-Piercy C, et al. The management of nausea and vomiting in pregnancy and hyperemesis gravidarum (Green-top Guideline No.69). BJOG. 2024;131:e1–30. 38311315 10.1111/1471-0528.17739

[9] Lowe S, et al. Guideline for the management of nausea and vomiting in pregnancy and hyperemesis gravidarum. Society of Obstetric Medicine of Australia and New Zealand; 2019.

[10] Boelig RC, et al. Interventions for treating hyperemesis gravidarum. Cochrane Database Syst Rev. 2016;5:CD010607. 10.1002/14651858.CD010607.pub2 PMC10421833 27168518

[11] Lowe SA, Steinweg KE. Review article: management of hyperemesis gravidarum and nausea and vomiting in pregnancy. Emerg Med Australas. 2022;34(1):9–15. 34872159 10.1111/1742-6723.13909

[12] Australian Institute of Health and Welfare. Australia's mothers and babies. 2025; Available from: https://www.aihw.gov.au/reports/mothers-babies/australias-mothers-babies/contents/antenatal-period/antenatal-visits. Cited 2025 13th November.

[13] Freeman N, et al. Exploring midwifery role and scope in acute early pregnancy care: a survey of midwives and midwifery students in Australia. BMC Pregnancy Childbirth. 2025;25(1):458. 40240973 10.1186/s12884-025-07567-3 PMC12004735

[14] Hsiao HF, et al. Pregnant women report being denied medications to treat severe nausea and vomiting of pregnancy or hyperemesis gravidarum - findings from an Australian online survey. Aust N Z J Obstet Gynaecol. 2021;61(4):616–20. 33984156 10.1111/ajo.13359

[15] Raymond SH. A survey of prescribing for the management of nausea and vomiting in pregnancy in Australasia. Aust N Z J Obstet Gynaecol. 2013;53(4):358–62. 23346891 10.1111/ajo.12045

[16] Gadsby R, et al. Nausea and vomiting of pregnancy and resource implications: the NVP Impact Study. Br J Gen Pract. 2019;69(680):e217–23. 30559108 10.3399/bjgp18X700745 PMC6400600

[17] Nana M, et al. Hyperemesis gravidarum in the primary care setting: cross-sectional study of GPs. BJGP Open. 2022;6(1). 10.3399/BJGPO.2021.0119. 10.3399/BJGPO.2021.0119 PMC8958745 34620600

[18] Baggley A, et al. Determinants of women’s decision making on whether to treat nausea and vomiting of pregnancy pharmacologically. J Midwifery Womens Health. 2004;49(4):350–4. 15236716 10.1016/j.jmwh.2004.03.011

[19] Fiaschi L, et al. Clinical management of nausea and vomiting in pregnancy and hyperemesis gravidarum across primary and secondary care: a population-based study. BJOG. 2019;126(10):1201–11. 30786126 10.1111/1471-0528.15662

[20] Trovik J, Vikanes A. Hyperemesis gravidarum is associated with substantial economic burden in addition to severe physical and psychological suffering. Isr J Health Policy Res. 2016;5:43. 27766142 10.1186/s13584-016-0099-y PMC5056484

[21] Evans L, et al. Scoping review: exploring the equity impact of current digital health design practices. Oxf Open Digit Health. 2023;1:1–15.

[22] van den Heuvel J, et al. eHealth as the next-generation perinatal care: an overview of the literature. J Med Internet Res. 2018;20(6):e202. 29871855 10.2196/jmir.9262 PMC6008510

[23] Petherbridge RL, et al. Outpatient models of care for pregnant women with hyperemesis gravidarum: a scoping review. Br J Midwifery. 2025;33(4):208–19.

[24] Walker P, et al. A scoping review of models of care and services for nausea and vomiting in pregnancy and hyperemesis gravidarum. BMC Pregnancy Childbirth. 2025;25(1):1012. 41044770 10.1186/s12884-025-08093-y PMC12495835

[25] The Royal Hospital for Women. MotherSafe. 2024; Available from: https://www.seslhd.health.nsw.gov.au/royal-hospital-for-women/services-clinics/directory/mothersafe. Cited 2024 27th November.

[26] NSW Health. Nausea and vomiting in pregnancy and hyperemesis gravidarum. NSW: NSW Health; 2022.

[27] Ebrahimi N, et al. Nausea and vomiting of pregnancy: using the 24-hour Pregnancy-Unique Quantification of Emesis (PUQE-24) scale. J Obstet Gynaecol Can. 2009;31(9):803–7. 19941704 10.1016/S1701-2163(16)34298-0

[28] Benchimol E, et al. The REporting of studies Conducted using Observational Routinely-collected health Data (RECORD) Statement. PLoS Med. 2015;12(10):e1001885. 26440803 10.1371/journal.pmed.1001885 PMC4595218

[29] Tong A, Sainsbury P, Craig J. Consolidated criteria for reporting qualitative research (COREQ): a 32-item checklist for interviews and focus groups. Int J Qual Health Care. 2007;19(6):349–57. 17872937 10.1093/intqhc/mzm042

[30] Harris PA, et al. Research electronic data capture (REDCap)--a metadata-driven methodology and workflow process for providing translational research informatics support. J Biomed Inform. 2009;42(2):377–81. 18929686 10.1016/j.jbi.2008.08.010 PMC2700030

[31] Koren G, et al. Motherisk-PUQE (pregnancy-unique quantification of emesis and nausea) scoring system for nausea and vomiting of pregnancy. Am J Obstet Gynecol. 2002;186(5 Suppl Understanding):S228–31. 12011891 10.1067/mob.2002.123054

[32] O’Brien EC, et al. The IRIS clinic: A Protocol for a mixed-methods study evaluating the management of Hyperemesis Gravidarum. Contemp Clin Trials Commun. 2024;39:101227. 39007106 10.1016/j.conctc.2023.101227 PMC11240289

[33] Sekhon M, Cartwright M, Francis JJ. Acceptability of healthcare interventions: an overview of reviews and development of a theoretical framework. BMC Health Serv Res. 2017;17(1):88. 28126032 10.1186/s12913-017-2031-8 PMC5267473

[34] Beecher C, et al. Measuring women’s experiences of maternity care: a systematic review of self-report survey instruments. Women Birth. 2021;34(3):231–41. 32522442 10.1016/j.wombi.2020.05.002

[35] Bureau of Health Information. Maternity Care Survey. 14 June 2023]; Available from: https://www.bhi.nsw.gov.au/nsw_patient_survey_program/maternity_care_survey.

[36] Braun V, Clarke V. Thematic analysis: a practical guide. Sage; 2021.

[37] Dhakal K. NVivo. J Med Libr Assoc. 2022;110(2):270–2. 10.5195/jmla.2022.1271. 10.5195/jmla.2022.1271 PMC9014916 35440911

[38] Australian Bureau of Statistics. Remoteness Structure. Jul2021-Jun2026. Available from: https://www.abs.gov.au/websitedbs/D3310114.nsf/home/remoteness+structureCited 2024 31st October.

[39] HealthStats NSW. Age of women at the time of giving birth (2022). 2022. Available from: https://healthstats.nsw.gov.au/indicator?name=-mab-age-mums-pdc&location=NSW&view=BarHorizontal&measure=Percent&groups=Period,Age%20(years)&compare=Age%20(years),Period&filter=Age%20(years),All%20ages,14%20years%20and%20under,25-29%20years,30-34%20years,35-39%20years,40-44%20years,45%20years%20and%20over,15-19%20years,20-24%20years&filter=Period,2022. Cited 2024 17th September.

[40] HealthStats NSW. Mothers giving birth in NSW by Remoteness category. 2023. Available from: https://www.healthstats.nsw.gov.au/indicator?name=-mab-total-mums-pdc&location=NSW&view=Trend&measure=Percent&groups=Remoteness%20category&compare=Remoteness%20category&filter=Remoteness%20category,NSW,Outer%20regional%20and%20remote,Inner%20regional,Major%20cities. Cited 2026 12th February.

[41] SickKids. Statement regarding closure of Motherisk Helplines. 2019. Available from: https://www.sickkids.ca/en/news/archive/2019/statement-regarding-closure-of-motherisk-helplines-/. Cited 2025 11th June.

[42] Madjunkova S, Maltepe C, Koren G. The leading concerns of American women with nausea and vomiting of pregnancy calling Motherisk NVP helpline. Obstet Gynecol Int. 2013;2013:752980. 23690784 10.1155/2013/752980 PMC3649700

[43] Madjunkova S, et al. Patterns of antiemetic use among American women with nausea and vomiting of pregnancy. Obstet Gynecol. 2014;123(SUPPL. 1):155S.

[44] Truong MBT, et al. The effect of a pharmacist consultation in early pregnancy on pregnant women’s quality of life: an intervention study. Pharmacoepidemiol Drug Saf. 2020;29(SUPPL 3):541–2.

[45] Ngo E, et al. Impact of a mobile application for tracking nausea and vomiting during pregnancy (NVP) on NVP symptoms, quality of life, and decisional conflict regarding NVP treatments: MinSafeStart randomized controlled trial. JMIR Mhealth Uhealth. 2022;10(7):e36226. 35787487 10.2196/36226 PMC9297140

[46] van Vliet R, et al. Patient Preferences and Experiences in Hyperemesis Gravidarum Treatment: A Qualitative Study. J Pregnancy. 2018;2018:5378502. 30515329 10.1155/2018/5378502 PMC6234451

[47] Frawley J, et al. Health care utilisation of women who experience pregnancy-related reflux, nausea and/or vomiting. J Matern Fetal Neonatal Med. 2017;30(16):1938–43. 27594351 10.1080/14767058.2016.1232711

[48] Doherty J, et al. Women's experiences of Hyperemesis Gravidarum (HG) and of attending a dedicated multi-disciplinary hydration clinic. Women and Birth. 2023;36(6):e661–8. 10.1016/j.wombi.2023.06.005. 10.1016/j.wombi.2023.06.005 37438233

[49] Beirne ER, et al. The far-reaching burden of Hyperemesis Gravidarum - an exploration of women's experiences and perceptions of healthcare support. Women Health. 2023;63(7):1–10. 10.1080/03630242.2023.2219749. 10.1080/03630242.2023.2219749 37334442

[50] Elder TJ, Iacurto G, Deys L. Enhancing maternal wellbeing: a qualitative exploration of women’s experiences of tailored education and holistic support while experiencing hyperemesis gravidarum. Midwifery. 2025;141:104258. 39637727 10.1016/j.midw.2024.104258

[51] Geeganage G, et al. Emergency department burden of hyperemesis gravidarum in the United States from 2006–2014. Am J Obstet Gynecol Glob Rep. 2023;3:100166. 10.1016/j.xagr.2023.100166. PMC9975274 36876158

[52] Abedian Z, et al. The effect of telephone support on the severity of nausea and vomiting in the first trimester of pregnancy in the primiparous women. Iranian Journal of Obstetrics, Gynecology and Infertility. 2014;17(118):22–9.

[53] Abedian Z, et al. The effects of telephone support on stress and perceived social support in primiparous women experiencing nausea and vomiting in the first half of pregnancy. J Midwifery Reprod Health. 2015;3(2):328–34.

[54] Isbir GG, Mete S. The effect of counselling on nausea and vomiting in pregnancy in Turkey. Sex Reprod Healthc. 2016;7:38–45. 26826044 10.1016/j.srhc.2015.11.005

[55] Liu MC, et al. Effects of professional support on nausea, vomiting, and quality of life during early pregnancy. Biol Res Nurs. 2014;16(4):378–86. 24113384 10.1177/1099800413506036

[56] Chua-Gocheco A, et al. Summary of Motherisk calls for 2011. Birth Defects Res A Clin Mol Teratol. 2012;94(5):408.

[57] Maltepe C, et al. The effects of counseling and predictors of pregnancy outcomes in women with hyperemesis gravidarum. Obstet Gynecol. 2015;125(SUPPL. 1):101S.

[58] Godbole KG, et al. Experiences from Garbha-Swasthya helpline. Indian J Public Health. 2015;59(2):149–52. 26021655 10.4103/0019-557X.157538

[59] Patil AS, et al. Health care providers’ use of a drug information service for pregnancy-related inquiries. J Am Pharm Assoc 2003. 2014;54(5):502–9. 25216880 10.1331/JAPhA.2014.13093

[60] Madjunkova S, Maltepe C, Koren G. The leading concerns of American women with nausea and vomiting of pregnancy (NVP) calling Motherisk NVP helpline. Birth Defects Res A Clin Mol Teratol. 2013;97(5):367. 10.1155/2013/752980 PMC3649700 23690784

[61] Lim JM, Sullivan E, Kennedy D. MotherSafe: review of three years of counselling by an Australian teratology information service. Aust N Z J Obstet Gynaecol. 2009;49(2):168–72. 19432605 10.1111/j.1479-828X.2009.00976.x

[62] Kennedy D, et al. Review of calls to an Australian teratogen information service regarding psychotropic medications over a 12-year period. Aust N Z J Obstet Gynaecol. 2013;53(6):544–52. 24028467 10.1111/ajo.12129

[63] Grzeskowiak LE, Hill M, Kennedy DS. Phone calls to an Australian pregnancy and lactation counselling service regarding use of galactagogues during lactation - the MotherSafe experience. Aust N Z J Obstet Gynaecol. 2018;58(2):251–4. 29057459 10.1111/ajo.12731

[64] Hegedus E, et al. Calls to a major teratogen information service regarding exposures during breastfeeding. Breastfeed Med. 2019;14(9):674–9. 31368784 10.1089/bfm.2019.0010

[65] Ritchie HE, et al. Utilisation of a NSW teratology information service by pharmacists and patients referred by a pharmacist from 2000 to 2018. Aust N Z J Obstet Gynaecol. 2020;60(3):412–8. 31583698 10.1111/ajo.13071

[66] Ritchie HE, et al. A descriptive analysis of calls to the NSW Teratogen Information Service regarding use of anti-infectives during pregnancy. PLoS ONE. 2022;17(10):e0270940. 36201464 10.1371/journal.pone.0270940 PMC9536608

[67] Bispo Junior JP. Social desirability bias in qualitative health research. Rev Saude Publica. 2022;56:101. 10.11606/s1518-8787.2022056004164. 10.11606/s1518-8787.2022056004164 PMC9749714 36515303

[68] Wilson M, et al. Family physician access to specialist advice by telephone. Can Fam Physician. 2016;62:e668–76. 28661886 PMC9844567

