# Supplementary material for: The Australian MotherSafe enhanced service for nausea and vomiting in pregnancy and hyperemesis gravidarum: a mixed methods study
Source: BMC Pregnancy Childbirth. 2026 May 29;26:825. doi: 10.1186/s12884-026-09356-y (PMC13411966; doi:10.1186/s12884-026-09356-y)
Supplement: Supplementary file 1 — Supplementary Material 1: The MotherSafe enhanced NVP/HG Service. [file 12884_2026_9356_MOESM1_ESM.pdf]

Supplementary Material 1: The MotherSafe enhanced NVP/HG Service

MotherSafe Teratogen Information Service

The MotherSafe Teratogen Information Service has a well-established profile with consumers and health care providers, providing telephone advice across the state of New South Wales (NSW) in Australia since 2000. The phone service is able to be accessed through a local telephone number for NSW as well as a toll-free telephone number that does not incur any charges for the caller.

When women call the service, they speak with one of the telephone counsellors, who review the reason for the call and offer up to date evidence-based advice regarding the exposure in question. This is done in the context of timing in preconception, pregnancy and lactation. The service also offers a medical clinic for complex preconception counselling and exposures for women throughout NSW. This clinic requires a referral from a primary care or specialist medical practitioner.

MotherSafe Enhanced NVP/HG Service

The MotherSafe enhanced NVP/HG service was introduced into the existing MotherSafe service, in April 2023. The expansion of the service for NVP/HG had multiple components that are described below. The NVP/HG service initially offered extended hours from 5pm-9pm to enable calls to be made after work hours, however this was ceased from July 2024 following review of call numbers and staffing. Calls are now accepted during the routine 9am-5pm, Monday-Friday hours of the MotherSafe service.

Telephone counselling service

During a call to the NVP/HG service, women are assessed for their NVP symptoms over the previous 24 hours using the Pregnancy-Unique Quantification of Emesis and Nausea (PUQE-24) scale [1]. In addition, standardised questions assessing their clinical condition are asked, which address pregnancy context, a complete medical history and medications taken regularly. Questions are included to explore underlying conditions that contribute to the development of NVP/HG, such as thyroid disease.

Management advice offered aligns with the NSW guidelines for NVP/HG [2]. Other guidelines are also referenced including those from the Society of Obstetric Medicine Australia and New Zealand (SOMANZ) [3] and the HER Foundation [4]. Medications (onset of action and appropriate duration) are discussed with regards to the indication, dose, known side effects. Rationalisation of pregnancy supplements is discussed to prioritise folic acid and iodine replacement and consider thiamine as indicated. If the caller takes other regular medications then interactions with these are also discussed where relevant. The scope of medications discussed includes those relevant for nausea and vomiting, gastro-oesophageal reflux disease, sleep and bowel management.

A dietary review is carried out that quantifies oral intake of food and fluids, as well as assessing weight change during the pregnancy. Recommendation of access to intravenous fluids is made through the known pathways at each local health district (n=15). For relevant calls, consideration of thromboprophylaxis is discussed, usually in the context of those admitted as an inpatient if this had not already been discussed. For calls related to inpatient care, follow up with the healthcare provider occurs as the context requires. A mental health assessment using the K-10 and NVPQOL depression score is performed if women report impacts on their mood from their symptoms.

Standard operating procedures were created for the service that outline escalation pathways for women that call with signs of deterioration requiring acute management. Women with a PUQE score of ≥13, those describing signs of acute deterioration or acute complications of excessive nausea and vomiting such as haematemesis, significant weight loss and restriction of oral intake, and those with underlying diagnoses that

exposed them to other deterioration risks such as type 1 diabetes mellitus, are advised to attend acute care services such as their GP or the Emergency Department dependent on their location and severity of symptoms. Where there are concerns regarding escalation, this is discussed with the medical team at MotherSafe. Referral to the service psychologist is offered for women that report a PUQE of ≥13, have an underlying mental health diagnosis, self-report impacts on mood, or where the call raises concerns regarding the impact of NVP/HG on the individual. Clients are offered up to 6 appointments with the psychologist as required, with the long-term aim of linking them with the services offered through their local health district to continue support during pregnancy as required.

Follow up

All women are offered routine follow up calls at 48-72 hours after the initial call (with consent). Women can decline this option. Women are called up to 3 times on the designated phone number provided, with messages left for unanswered calls where possible. Calls are made at the time suggested by the caller as convenient, or at different times in the day to help cater to caller availability. Some women who call have multiple follow-up calls for the one initial call, which are each recorded as a follow up call in the database.

The follow up call includes a follow-up symptom review, discussion of any medication changes and their perceived efficacy, review for side effects and repeat measurement of the PUQE-24 scale to compare with the previous reported score. During the call, attendance at other healthcare providers is also discussed, including the outcomes of this. If women were not able to access appropriate management within their residential area, an additional follow up call is offered if there is need for it. For women without response to the medication regime, an option for a telehealth appointment with an obstetrician or obstetric physician within the NVP/HG service is discussed. If women are satisfied with their response to medications and linked with ongoing care, then no further follow up calls were conducted. Women are reminded that they can contact the service at any time. All call data is logged within a database.

Service referral mapping

The public health system in NSW is divided into local health districts (n=15) that have corresponding maternity and newborn services. As the NVP/HG service covered the whole state, escalation pathways were needed for the public health system in each area. Escalation is required for women who call that need expedited medical review. As the service delivery design is different for each health district, the entry pathway for women to be treated also requires clarification. MotherSafe NVP/HG staff liaise with representatives from each LHD to accurately record service access and referrals for women. Some women are routed via emergency department services for initial review, and others access services through different parts of maternity services. Timing of presentation also influences service availability at each site. Women accessing pregnancy care through the private health system also have the option to escalate care to their chosen obstetrician.

References

1. Ebrahimi, N., et al., *Nausea and vomiting of pregnancy: using the 24-hour Pregnancy-Unique Quantification of Emesis (PUQE-24) scale.* J Obstet Gynaecol Can, 2009. **31**(9): p. 803-807.
2. NSW Health, *Nausea and Vomiting in Pregnancy and Hyperemesis Gravidarum*. 2022, NSW Health: NSW.
3. Lowe, S., et al., *Guideline for the management of nausea and vomiting in pregnancy and hyperemesis gravidarum*. 2019, Society of Obstetric Medicine of Australia and New Zealand.
4. HER Foundation, *HG Treatment Protocol: Hyperemesis Gravidarum (HG) Management Protocol*. 2019, accessed online 25th April 2026, https://www.hyperemesis.org/tools/treatment-protocol/
