# Supplementary material for: The Australian MotherSafe enhanced service for nausea and vomiting in pregnancy and hyperemesis gravidarum: a mixed methods study
Source: BMC Pregnancy Childbirth. 2026 May 29;26:825. doi: 10.1186/s12884-026-09356-y (PMC13411966; doi:10.1186/s12884-026-09356-y)
Supplement: Supplementary file 2 — Supplementary Material 2: Patient experience and acceptability interview guide. [file 12884_2026_9356_MOESM2_ESM.pdf]

Supplementary Material 2 (S2)

Patient experience and acceptability interview guide

Introduction

Thank you for agreeing to be interviewed as part of this evaluation of the Hyperemesis Gravidarum Initiative.

The purpose of this interview is to explore your experiences with the MotherSafe NVP/HG service.

Throughout the interview I will be asking you to reflect on your experiences of the care and advice that you have received to support your pregnancy and management of nausea and vomiting, or HG. It would be great if you could be as detailed and specific as possible in your responses. There are no right or wrong answers and your responses will be kept anonymous and will only be reported as grouped findings combined with the experiences of other interview participants, so please feel free to speak openly about your experiences. We will value all of your comments and feedback, as this may help inform decision making around health care services for women with NVP/HG in the future.

This interview will take up to 30 minutes, but if you would like to stop at any point then please just let me know.

Is there anything else you'd like to know before we start?

I'd like to audio record our conversation to make sure I capture everything you say. Is that OK with you? [turn on recorder at this point if consent is given. If consent is not given to audio record, then ask if it is ok to take notes.]

And before we start, may I ask you to confirm that you understand the purpose of this interview and freely consent to take part?

Indicative interview questions

**Introduction and rapport building**	• Can you tell me briefly about yourself?<br>• Congratulations on your [*pregnancy*], would you mind telling me a bit about your pregnancy care journey? I'm also interested in understanding, aside from the MotherSafe NVP/HG service, if you have accessed any other services for support with nausea and/or vomiting in this pregnancy?
**Intervention Coherence –** The extent to which the participant understands the intervention and how it works	• In your own words, could you tell me about your understanding of the *MotherSafe NVP/HG* service?<br>• Could you also describe how you came to learn about this service and how you engaged with the *MotherSafe NVP/HG* service?<br>• Can you tell me about the type of care or support you received from the *MotherSafe NVP/HG* service?
**Self-efficacy –** The participant's confidence that they can perform the behaviour(s) required to participate in the intervention	• Do you recall having any hesitations or concerns about your ability to engage with the *MotherSafe NVP/HG* service?

**Burden –** The perceived amount of effort that is required to participate in the intervention	• Can you describe for me the process for accessing the *MotherSafe NVP/HG* service? How easy or difficult did you find this process?
**Opportunity costs –** The extent to which benefits, profits or values must be given up to engage in the intervention	• Thinking about the times when you used the service, did you have to make special arrangements to get access to the service? For instance, did you need to take time off work or make alternative arrangements for childcare?
**Affective attitude –** How an individual feels about the intervention<br><br>**Ethicality –** The extent to which the intervention has good fit with an individual's value system	In thinking now about the service that you received:<br>• Did you feel that it was a well organised and well-run service?<br>• What was the communication like between you and care providers? (Prompt: Did you understand the information they provided? Did you feel you had enough information?)<br>• Did you feel respected and comfortable asking questions?<br>• To what extent did you feel like the care provider(s) understood your situation and was able to provide relevant support? (Prompt: Did you have confidence and trust in the care provider/s?)
**Perceived effectiveness –** The extent to which the intervention is perceived as likely to achieve its purpose	• To what extent did you feel that the *MotherSafe NVP/HG* service met your care needs? (Prompt: Can you suggest any ways in which it could have helped you more?)<br>• When we talk about quality of life, we mean an overall view of your well-being, health, comfort and social participation. To what extent do you think that your quality of life was enhanced as a result of the *MotherSafe NVP/HG* service?<br>• What was it about the service that [*did/did not*] support you in your pregnancy? *Prompt: What did you like and what did you dislike about this service?*<br>• Would you recommend the *MotherSafe NVP/HG* service to other women with NVP/HG?
**Suggested improvements**	• Do you have any suggestions for improving this service for women with NVP/HG?
**Closing and thank you**	• Is there anything else you would like to discuss? Do you have any questions for me?<br>• We will be getting the recording from this interview professionally transcribed. Would you like us to send you a copy of this document for your review?<br>• Thank you very much for your time today.
